# Listeria monocytogenes Meningitis With Hydrocephalus in a Patient With Sjögren’s Syndrome and Penicillin Allergy

**DOI:** 10.7759/cureus.105527

**Published:** 2026-03-19

**Authors:** Xin Ying Chong, Noor Nadia Binti Sarbini, Chee Yik Chang

**Affiliations:** 1 Internal Medicine, Hospital Sultanah Aminah, Johor Bahru, MYS; 2 Infectious Diseases, Hospital Sultanah Aminah, Johor Bahru, MYS

**Keywords:** acute hydrocephalus, bactrial meningitis, listeria monocytogens, penicillin allergy, sjogren’s syndrome

## Abstract

*Listeria monocytogenes* is an opportunistic Gram-positive intracellular pathogen that predominantly affects elderly and immunocompromised individuals. Central nervous system involvement may result in meningitis or meningoencephalitis, with hydrocephalus representing a rare but serious complication associated with high morbidity and mortality. This case report describes a 62-year-old woman with Sjögren’s syndrome on methotrexate who presented with fever, headache, and altered mental status. Imaging revealed communicating hydrocephalus, and CSF analysis confirmed *Listeria monocytogenes* meningitis via multiplex PCR. Management was complicated by penicillin allergy and subsequent hypersensitivity to trimethoprim-sulfamethoxazole, eventually requiring a transition to oral linezolid. Targeted therapy led to the resolution of hydrocephalus and significant neurological recovery by discharge. This case highlights the importance of considering *Listeria* in immunocompromised patients and the necessity of alternative antimicrobial strategies when faced with rare complications and complex drug allergies.

## Introduction

*Listeria monocytogenes* is a Gram-positive, facultative intracellular anaerobic bacillus. It is primarily transmitted via the fecal-oral route through contaminated food, including unpasteurized dairy products, raw vegetables, and raw or undercooked meat and poultry [1].

*Listeria monocytogenes* commonly causes severe infections in high-risk groups such as the elderly, neonates, pregnant women, and immunocompromised individuals. In contrast, infection in immunocompetent individuals is uncommon and typically presents as a mild, self-limited gastroenteritis. In immunocompromised patients, invasive listeriosis may manifest as meningitis, meningoencephalitis, brain abscess, endocarditis, septic arthritis, osteomyelitis, and liver abscess. Listeria meningitis typically presents with fever, headache, neck stiffness, altered mental status, and focal neurological deficits [1,2]. A surveillance study in the Netherlands of 375 cases of Listeria meningitis found that the peak incidence rates were in neonates (0.61 per 100,000 live births) and elderly persons (peak at 87 years; 0.53 cases per 100,000 population of the same age) [3].

Hydrocephalus is a recognized complication of* Listeria monocytogenes* meningoencephalitis and is associated with significant morbidity and mortality [4,5]. Listeria meningitis has been reported in patients with connective tissue disease and immunosuppressive medication [6]. To our knowledge, no case of Listeria meningitis in a patient with Sjögren’s syndrome has been documented. Here, we report a case of *Listeria monocytogenes* meningitis in a patient with Sjögren’s syndrome receiving immunosuppressive therapy.

## Case presentation

A 62-year-old woman with underlying Sjögren’s syndrome on hydroxychloroquine and weekly methotrexate presented with a two-week history of intermittent fever, headache, and lethargy, followed by two days of altered mental status prior to admission. She had documented allergies to etoricoxib (Arcoxia) and ampicillin. She denied cough, vomiting, diarrhea, abdominal pain, head injury, seizures, or urinary and bowel incontinence. There was no history of recent travel or sick contact. She initially sought treatment at a private clinic and received oral antibiotics; however, her symptoms persisted.

Upon presentation to our hospital, she was confused with a Glasgow Coma Scale (GCS) score of E4V4M5. Her blood pressure was 134/70 mmHg, pulse rate 56 beats per minute, temperature 37°C, respiratory rate 22 breaths per minute, and oxygen saturation 98% on 3 L/min oxygen via nasal prongs. Neurological examination revealed horizontal nystagmus, bilateral diplopia, neck stiffness, and generalized limb weakness.

Laboratory investigations showed leucocytosis with a white blood cell count of 18 × 10³/µL (normal range = 4-10 × 10³/µL), hemoglobin of 11 g/dL (normal range = 12-16 g/dL), platelet count of 222 × 10³/µL (normal range = 150-400 × 10³/µL), and markedly elevated C-reactive protein of 300 mg/L (normal < 5 mg/L). Renal and liver function tests were within normal limits. Blood cultures, HIV, hepatitis B, hepatitis C, and venereal disease research laboratory (VDRL) serology were negative (Table 1). 

**Table 1:** Laboratory findings on admission

Parameters	Patient values	Reference range
Haemoglobin	11 g/dL	12–16 g/dL
White blood cell	18 × 10³/µL	4–10 × 10³/µL
Platelet	222 × 10³/µL	150–400 × 10³/µL
C-reactive protein	300 mg/L	< 5 mg/L

Computed tomography (CT) of the brain demonstrated communicating hydrocephalus without cerebral oedema. A diagnostic lumbar puncture revealed an opening pressure of 13 cmH₂O. The cerebrospinal fluid (CSF) was clear and colourless, with glucose 0.5 mmol/L (normal range = 2.5-4.4 mmol/L), protein 4.54 g/L (normal range = 0.15-0.45 g/L), and 558 white blood cells/µL (normal range = 0-5 cells/µL) (Table 2). CSF India ink stain and acid-fast bacilli smear were negative. Empirical treatment with intravenous meropenem and acyclovir was initiated pending results of the meningitis/encephalitis multiplex PCR panel.

**Table 2:** CSF findings on Days 1 and 4 of admission.

Parameters	Day 1	Day 4	Reference range
Opening pressure (cmH₂O)	13	12	10 - 12
CSF Glucose (mmol/L)	0.5	5.66	2.5–4.4
CSF Protein (g/L)	4.54	5.66	0.15–0.45
CSF cell count (wbc/ µL)	588	1250	0 - 5
CSF culture	No growth	No growth	
CSF multiplex PCR	Listeria monocytogenes	Not done	

**Figure 1:**
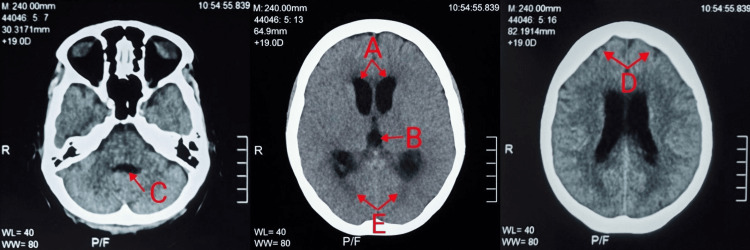
CT brain imaging revealed dilated bilateral lateral ventricles (A) and third ventricles (B), alongside a prominent fourth ventricle (C). There was associated effacement of the bilateral cerebral sulci (D), suggesting communicating hydrocephalus with concurrent cerebral oedema. Periventricular hypodensities were also noted, likely representing transependymal CSF seepage (E).

On day 3 of hospitalization, her neurological status deteriorated to GCS E3V2M4. Repeat CT brain demonstrated worsening hydrocephalus with CSF seepage and cerebral oedema (Figure 1). On day 4, the CSF multiplex PCR returned positive for *Listeria monocytogenes* DNA, confirming the diagnosis of Listeria meningitis. In view of her ampicillin allergy, antimicrobial therapy was switched to intravenous trimethoprim-sulfamethoxazole (10 mg/kg/day based on trimethoprim component).

**Figure 2:**
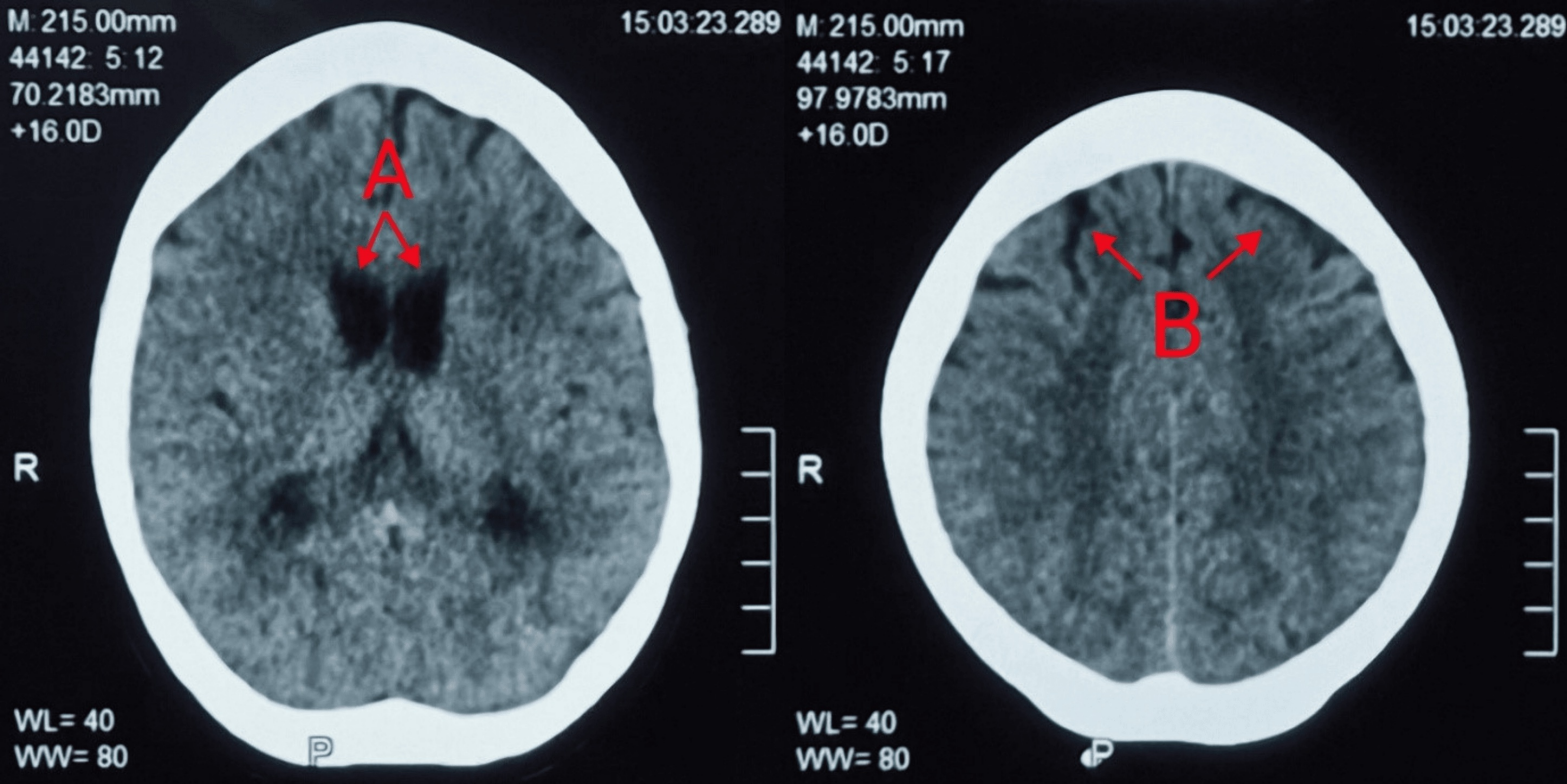
CT brain imaging revealed a marked reduction in ventricular dilatation and the resolution of previously noted periventricular hypodensities (A). The bilateral cerebral sulci are no longer effaced (B), indicating the resolution of both the communicating hydrocephalus and the associated cerebral oedema.

Due to persistent clinical concern, a repeat lumbar puncture was performed, revealing CSF glucose 1.1 mmol/L, protein 5.66 g/L, and 1,250 white blood cells/µL (54% polymorphonuclear cells). CSF culture showed no growth. By day 6, her level of consciousness improved significantly, with resolution of nystagmus and diplopia. Repeat CT brain demonstrated resolution of hydrocephalus and cerebral oedema (Figure 2).

However, on day 10, she developed a generalized pruritic maculopapular rash consistent with trimethoprim-sulfamethoxazole hypersensitivity. Antimicrobial therapy was therefore changed to oral linezolid 600 mg twice daily. She responded well to treatment and was discharged on day 20 of hospitalization with marked neurological recovery.

## Discussion

*Listeria monocytogenes* is a Gram-positive, facultative intracellular anaerobic bacillus transmitted primarily via contaminated food products, including unpasteurized dairy products, raw vegetables, and undercooked meat. It predominantly affects high-risk populations such as the elderly, neonates, pregnant women, and immunocompromised individuals [1,2].

Community-acquired bacterial meningitis remains a life-threatening condition despite advances in antimicrobial therapy. While *Streptococcus pneumoniae* and *Neisseria meningitidis* are the most common pathogens, *Listeria* *monocytogenes* represents an important cause in elderly and immunocompromised patients. Advanced age, diabetes mellitus, malignancy, solid organ transplantation, and prolonged immunosuppressive therapy are well-recognized risk factors [7,8]. In our patient, the presence of immunosuppressive therapy in the setting of primary Sjögren’s syndrome likely predisposed to invasive listeriosis.

Unlike typical extracellular meningeal pathogens, *L. monocytogenes* is a facultative intracellular organism that invades macrophages and epithelial cells, spreads via actin polymerization, and evades humoral immune responses. Therefore, effective host defence depends largely on intact cell-mediated immunity [9]. Patients with primary Sjögren’s syndrome exhibit systemic immune dysregulation and impaired mucosal barrier function, which may increase susceptibility to opportunistic and intracellular pathogens [10]. Previous studies have demonstrated increased hospitalization rates for serious infections among patients with Sjögren’s syndrome, including infections caused by opportunistic organisms such as Listeria [11].

Clinically, Listeria meningitis presents similarly to other forms of bacterial meningitis, with fever, headache, neck stiffness, altered mental status, and focal neurological deficits. However, complications such as hydrocephalus have been reported and are associated with increased morbidity and mortality [4,5]. The development of hydrocephalus in our patient highlights the aggressive nature of central nervous system listeriosis in immunocompromised hosts.

CSF analysis remains the cornerstone of diagnosis. A positive CSF culture confirms the diagnosis and enables antimicrobial susceptibility testing. PCR is particularly useful in cases with negative Gram stain or culture and allows rapid identification of common meningeal pathogens, including *Listeria monocytogenes* [12].

First-line treatment for *Listeria monocytogenes* meningitis includes ampicillin, amoxicillin, or penicillin G. Trimethoprim-sulfamethoxazole and linezolid are alternative options in patients with β-lactam allergy [13,14]. Although the optimal duration of therapy has not been definitively established, current guidelines recommend at least 21 days of treatment, with longer courses considered in complicated cases [13].

Management of this case was particularly challenging due to the patient’s documented ampicillin allergy, as β-lactam antibiotics such as ampicillin or penicillin G are the first-line treatment for *Listeria monocytogenes* meningitis. The need to avoid standard therapy limited initial antimicrobial options and required the use of alternative agents with less robust clinical data. Although intravenous trimethoprim-sulfamethoxazole is an established alternative, the patient subsequently developed a hypersensitivity reaction, necessitating a further switch to linezolid.

## Conclusions

This case highlights the importance of considering *Listeria monocytogenes* as a potential pathogen in immunocompromised patients presenting with meningitis, particularly those receiving immunosuppressive therapy. Early recognition, prompt initiation of appropriate antimicrobial therapy, and close monitoring for complications such as hydrocephalus are crucial to improving clinical outcomes.
